# Seasonal Variations and Resilience of Bacterial Communities in a Sewage Polluted Urban River

**DOI:** 10.1371/journal.pone.0092579

**Published:** 2014-03-25

**Authors:** Tamara García-Armisen, Özgül İnceoğlu, Nouho Koffi Ouattara, Adriana Anzil, Michel A. Verbanck, Natacha Brion, Pierre Servais

**Affiliations:** 1 Ecology of Aquatic Systems, Université Libre de Bruxelles, Campus de la Plaine, Brussels, Belgium; 2 Department of Water Pollution Control, Université Libre de Bruxelles, Campus Plaine, Brussels, Belgium; 3 Analytical and Environmental Chemistry, Vrije Universiteit Brussels, Brussels, Belgium; The University of Hong Kong, Hong Kong

## Abstract

The Zenne River in Brussels (Belgium) and effluents of the two wastewater treatment plants (WWTPs) of Brussels were chosen to assess the impact of disturbance on bacterial community composition (BCC) of an urban river. Organic matters, nutrients load and oxygen concentration fluctuated highly along the river and over time because of WWTPs discharge. Tag pyrosequencing of bacterial 16S rRNA genes revealed the significant effect of seasonality on the richness, the bacterial diversity (Shannon index) and BCC. The major grouping: -winter/fall samples versus spring/summer samples- could be associated with fluctuations of *in situ* bacterial activities (dissolved and particulate organic carbon biodegradation associated with oxygen consumption and N transformation). BCC of the samples collected upstream from the WWTPs discharge were significantly different from BCC of downstream samples and WWTPs effluents, while no significant difference was found between BCC of WWTPs effluents and the downstream samples as revealed by ANOSIM. Analysis per season showed that allochthonous bacteria brought by WWTPs effluents triggered the changes in community composition, eventually followed by rapid post-disturbance return to the original composition as observed in April (resilience), whereas community composition remained altered after the perturbation by WWTPs effluents in the other seasons.

## Introduction

Rivers flowing through cities are often used as receiving body for treated and untreated urban wastewaters all over the world [1], [2]. Some of these sewage-contaminated rivers are amongst the most extreme examples of ecosystems disturbed by human activities. The impact of wastewater release on the receiving river depends primarily on the size of the city, the type of treatment applied to wastewaters and the flow of the river; the higher the river flow is, the higher its dilution capacity is. The UN predicted that, for 2050, 70% of the world population will be living in cities and this urban growth will mainly occur in less developed countries, where wastewater treatment facilities are scarce [2]. By 2025, it is expected that 27 megacities of more than 10 million inhabitants will exist, 21 of which in less developed countries [2]. Considering the importance, scarcity and fragility of freshwater ecosystems, research about the impact of the release of high amount of sewage on rivers functioning and ecological health is essential to preserve and have a rational management of this resource over a long term period.

It is now well known that microbial community inhabiting aquatic systems are one of the key players in the biogeochemical cycling of organic matter and nutrients and thus in the recovery and maintenance of ecosystem health and balance [3], [4]. Sewage brings high loads of organic and inorganic pollutants that strongly modify the habitat of native freshwater bacteria. For instance, basic parameters such as temperature, pH and dissolved oxygen which are known as drivers of the bacterial community composition (BCC) [5], [6], [7] in aquatic systems can be influenced by urban wastewater discharges. Additionally, the loads of toxic trace metals from industries, of Persistent Organic Pollutants (POPs) and pharmaceuticals resulting from human activities could also affect the BCC [8], [9], [10], [11]. Additionally, high concentrations of particulate matter within wastewater can modify light penetration in the water column and prevent phototrophic organisms’ growth.

Sewage also brings high loads of allochthonous microorganisms [12]. If the allochthonous bacteria remain active after their release in the environments, as it has been previously suggested [13], [14], [15], they can have a major impact on the BCC of the river and could influence biogeochemical cycles. Besides, utilization of waters contaminated with allochthonous pathogenic microorganisms causes health risk.

Investigating the microbial population dynamic and composition of sewage polluted rivers and evaluating the resistance (insensitivity to disturbance) and resilience (the rate of recovery after disturbance (of microbial communities are essential for understanding how ecosystem health and functioning could be affected by the disturbances related to major sewage discharges. Despite the importance of these ecosystems and the increasing interest and advances in freshwater microbial ecology [16], [17], [18], [19], papers devoted to BCC of urban rivers are still underrepresented in the scientific literature. Until now, the majority of the studies dealing with microbial community in urban aquatic settings have focused on indicator bacteria related to fecal and organic pollution [20], [21], [22], [23]. Although some studies were done on polluted river sediments [24],[25],[26],[27], to the best of our knowledge only few papers have examined the BCC of urban rivers waters [7], [28], [29], [30].

The objectives of the present study were thus: **(1)** to study the spatial and temporal variations of the BCC along a sewage polluted urban river; **(2)** to identify the main environmental factors driving the microbial population dynamics in the watercourse; **(3)** to compare the river BCC before and after the discharge of major wastewater treatment plants (WWTPs) effluents; and **(4)** to analyze the fate and dynamics of different functional groups of sewage related bacteria once discharged into the river.

For this purpose, the Zenne River in Brussels (Belgium), a river highly disturbed by urban wastewater, was chosen as a model. This small river, with an annual average discharge of 4 m^3^ s^−1^ upstream from Brussels, crosses the city and receives the effluents released by the two WWTPs of Brussels (treating together more than 1.5 million inhabitant equivalents); the average flow of treated wastewaters is in the same order of magnitude than the Zenne River flow rate upstream from Brussels. The river waters downstream from Brussels are thus roughly half composed of treated wastewaters, this proportion being even higher during the low flow periods of the river [31]. Seven stations along the river and the treated effluents of the two Brussels’s WWTPs were sampled during four sampling campaigns at different seasons in 2010. The BCC was analyzed using 16S rRNA gene amplicon 454 pyrosequencing; in parallel, a large set of environmental parameters were measured.

## Materials and Methods

### Ethics Statement

No specific ethical or institutional permits were required to sample the Zenne River and the experimental studies did not involve endangered or protected species. We also obtained permission from Vivaqua (www.vivaqua.be) to sample the effluent of Brussels South WWTP and from Aquiris (www.aquiris.be) to sample the effluent of Brussels North WWTP.

### Study Site and Sampling Strategy

**Figure 1:**
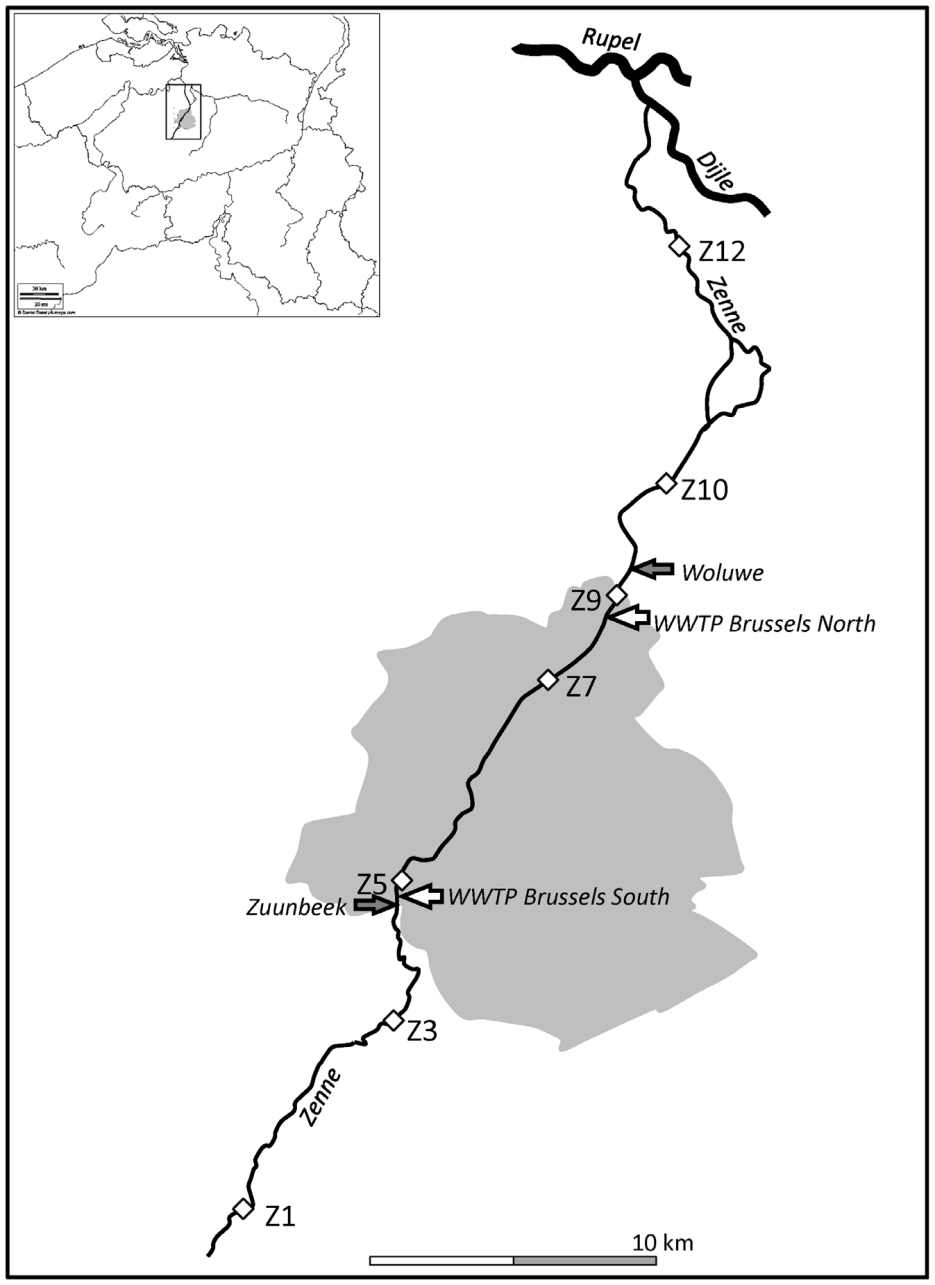
Map of the Zenne drainage network. Location of the sampling stations and the two Brussels WWTPs are represented.

The Zenne River is located in the Scheldt watershed and flows in the three Belgian regions (Wallonia, Flanders and Brussels-Capital); it is a tributary of the Dijle River (Fig. 1). The Zenne watershed (surface 991 km^2^) is characterized by agricultural activities in its upstream part and an important urbanization in its downstream part. The annual average discharge of the Zenne River arriving in Brussels from the upstream part of the watershed is 4 m^3^ s^−1^ (deduced from the last 20 years water flow data monitored by the Hydrologisch Informatie Centrum – HIC). Before the river reaches the Brussels area, it already receives several effluents of small scale WWTPs. The population density in the watershed is very high (on average 1260 inhabitants km^−2^) and mostly located in Brussels city and suburbs. The Zenne River has a total length of 103 km and crosses Brussels city from South to North over a distance of about 20 km. For sanitation purposes, the Zenne River was covered in the 19th century (1867–1871) in most of the stretch located in the Brussels region. In Brussels area, the Zenne River receives the sewage waters from two large WWTPs: the Brussels South WWTP (360,000 equivalent-inhabitants) in operation since the year 2000 and the Brussels North WWTP (1.2 million equivalent-inhabitants) in operation since 2007. The Zenne River also receives waters from two tributaries around the Brussels region the Zuunbeek, and the Woluwe (Fig. 1). Some small tributaries located in the Brussels area are diverted in the sewer collectors so that their waters reach Brussels WWTPs.

The two Brussels WWTPs function according to different technologies. The Brussels South WWTP treatment line includes a primary settling stage and a secondary biological treatment (activated sludge). At Brussels North WWTP, there are two treatment lines. The first one (biological line) includes a primary settling stage followed by a modern tertiary treatment technology (simultaneous removal of biodegradable organic carbon, nitrogen and phosphorus by an activated sludge process). The other treatment line (rain line) runs in parallel to the biological line when the discharge reaching the WWTP is too high in wet weather situations; this rain line uses only a coagulation-assisted high-rate lamellar settling process. On an annual basis, the volume treated in the biological line accounts for roughly 90% of the total volume reaching the WWTP.

Four sampling campaigns were performed in 2010 (January, April, July and October). Overall these sampling campaigns took place during marked dry weather periods; the discharge of the Zenne River just upstream from Brussels was always low during these campaigns:, 2.5 m^3^ s^−1^ in January 2010, 1.4 m^3^ s^−1^ in April 2010 and 2.0 m^3^ s^−1^ in July 2010, 2.9 m^3^ s^−1^ in October 2010. Comparatively, the April campaign was characterized by the driest conditions, both upstream and downstream Brussels city. Regarding October, there was actually a small rainfall occurring in Central Belgium during the sampling campaign but this did not even cause combined sewer overflow (CSO) discharge in the Zenne.

Seven stations were sampled along the Zenne River (Fig. 1) in the stretch located downstream from the confluence with its major right-bank tributary the Sennette. Accordingly, a kilometric scale along the longitudinal course of the river was defined; it is arbitrarily set at zero at Lembeek (station Z1) and increases from upstream to downstream. Stations Z1 (0 km) and Z3 (13 km) are located upstream from Brussels. Station Z5 (20 km) is located just downstream from the Brussels South WWTP effluents release. Stations Z7 (30 km) and Z9 (34 km) are located upstream and downstream from the Brussels North WWTP, respectively. Stations Z10 (39 km) and Z12 (51 km) are significantly downstream from the Brussels conurbation area. Besides, in each of the sampling campaigns, the effluents of both Brussels WWTPs were sampled (average daily samples collected with refrigerated automatic samplers) and analyzed.

### Physico-chemical Analysis and Bacterial Enumeration

Temperature, pH, and electrical conductivity (EC) were measured directly on site using a portable WTW 340 multiprobe. Dissolved oxygen was measured on the spot with a WTW oxi 323 field probe. Total alkalinity was determined by titration with HCl. Inorganic nitrogen (NH_4_, NO_2_ and NO_3_) and phosphorus (PO_4_) concentrations were determined by automatic colorimetric methods using a QuAAtro (Seal, Analytical) segmented flow analyzer system. Suspended particulate matter (SPM) was estimated as the weight of material retained on Whatman GF/F glass fiber filter (diameter 4.7 cm, particle retention size 0.7 μm) per volume unit after drying the filter at 105°C. For dissolved (DOC) and particulate organic carbon (POC) analysis, samples were filtered on GF/F glass fiber filters previously combusted at 550°C. DOC was measured in the filtrate while the material retained on the filter was used to estimate POC. Glassware receiving the water samples for DOC analysis was muffled at 550°C for 4 h after cleaning; samples for DOC analysis were preserved with H_3_PO_4_ (0.1% final concentration). DOC concentrations were measured with a total organic carbon analyzer in which inorganic carbon is eliminated by bubbling in the presence of phosphoric acid and organic carbon is oxidized by UV promoted persulfate oxidation and the produced CO_2_ detected by infrared spectrometry (Hiper-TOC, Thermo). The GF/F filters with the particulate material were first acidified to remove inorganic carbon. POC was analyzed with an elemental analyzer (Flash EA 1112, Thermo) where organic material is oxidized by catalytic flash combustion at 1020°C and the produced CO_2_ is measured by thermal conductivity. The DOC analyzer was calibrated using standard sucrose solutions, the POC analyzer was calibrated with acetanilide powder.

Bacterial abundance was determined by epifluorescence microscopy at 1000×magnification, after DAPI staining according to the procedure proposed by Porter and Feig [32].

### DNA Extraction

Microbial biomass was collected from water fraction and concentrate by filtration. An aliquot (from 0.8 L to 5.0 L) of each sample was filtered in triplicate directly through 0.2 μm pore-size, 47-mm-diameter polycarbonate filters (Millipore, MA). All filters were placed in 3 mL lysis buffer (50 mM Tris-HCl, pH 8.0, 40 mM EDTA, 750 mM sucrose) and stored at −20°C until use. DNA was extracted using a combination of enzymatic cell lysis and cetyltrimethyl ammonium bromide (CTAB) extraction protocol as previously described [33]. Dry DNA pellets were finally rehydrated in 100 μL of 10 mM Tris-HCl buffer (pH 7.4) and further purified using Min Elute reaction cleanup kit (QIAGEN). DNA concentration and purity were then determined using a Nanodrop ND-2000 UV-Vis spectrophotometer (Nanodrop, DE). Purified DNA extracts were stored at −20°C until use.

### Bacterial 16S rRNA Gene Tag-encoded FLX-titanium Amplicon Pyrosequencing

Bacterial tag-encoded FLX gene amplicon pyrosequencing (bTEFAP) analysis was carried out by means of a Roche 454 FLX instrument with titanium reagents. Titanium procedures were performed at the Research and Testing Laboratory (Lubbock, TX, USA) based upon RTL protocols (www.researchandtesting.com) as previously described [34], [35]. DNA samples were diluted to a final concentration of 20 ng/μL prior to bTEFAP. One-step PCR with a mixture of Hot Start and HotStar high-fidelity Taq polymerases was used. The PCR primers for FLX amplicon pyrosequencing were chosen to span the variable regions V1–V3 in the 16S rRNA gene: 27F (5′-GAGTTTGATCNTGGCTCAG-3′) and 519R (5′- GWNTTACNGCGGCKGCTG-3′) for *Bacteria*. These selected primers cover about 78% of publicly available 16S rDNA sequences of *Bacteria* (TestPrime tool at SILVA webpage http://www.arb-silva.de/search/testprime/). All sequences generated in this study can be downloaded from NCBI Short Read Archive, accession numbers: (SRP021937).

### 16S rRNA Data Processing

The Research and Testing pipeline (Lubbock, TX, USA) performed the denoising were performed on each region. Briefly data were decompressed and sequencing errors were reduced by trimming flows (i.e., denoising) and sequences by applying the following criteria: amplicons shorter than 200 bp in length, - Afterwards, using USEARCH [36], reads were dereplicated meaning they were clustered together into groups such that each sequence was an exact match to portion of the seed sequence for the cluster. Then, the seed sequence from each cluster was sorted by abundance, largest cluster to smallest cluster, without any minimum size restrictions on the clusters. Afterwards, putative chimeras (checked by using USEARCH [36]) were removed from our data set. Then singletons were also removed. The denoised and chimera checked reads were condensed into a fasta file. Then failed sequences and sequences that have low quality tags, primers, or ends were removed.

The open-source, platform-independent, community-supported software program, Mothur (Mothur v.1.25.1; http://www.mothur.org) was used to analyze the clean sequence data. We trimmed the barcodes and primers from the resulting sequences.

Rarefaction curves based on identified OTU, Shannon diversity index and richness estimator Chao1 were generated using Mothur for each sample at 97% similarity. Sequences were classified using the greengenes database at 80% confidence threshold with Mothur. After taxonomic assignment of the sequences down to the phylum, class and genus level, relative abundance of a given taxonomic group was set as the number of sequences affiliated with that group divided by the total number of sequences per sample.

### Statistical Analyses

Genera abundances were standardized, square root transformed, and assembled into a Bray Curtis similarity matrix to generate a multidimensional scaling (MDS) plot, using PRIMER 6 [37] and plotted to assess similarities among samples in two-dimensional space. MDS plots represent relative distances among samples in relation to the rank order of their relative similarities. The Spearman correlation test was used to analyze the correlations between relative abundance of genera and phylum/environmental parameters. The discrimination of bacterial assemblages based on water type was tested with one-way analysis of similarities (ANOSIM) using PRIMER 6 [37]. Similarity percentage (SIMPER) [38] was used to determine which genus contributed most to the dissimilarity between upstream stations (Z1, Z3), downstream stations (Z5, Z7, Z9, Z12 and WWTPs samples).

Rest of the analyses was conducted in R [39], using the vegan [40] and labdsv [41] packages. Procrustes analyses were done to determine the degree of concordance between MDS ordinations obtained at different taxonomic resolution levels (phylum, genera and OTU_0.03_) on R [39]. Overall patterns in the microbiome were illustrated using heatmaps. When constructing the heatmaps, differences among sites were characterized using Bray-Curtis distances and differences among taxa were characterized using Euclidean distances. In addition, data within samples were standardized to facilitate comparisons. Species that occurred only once were removed for clustering analysis and only those having a relative abundance of more than 5% were shown in the heatmaps. We quantified the common and unique influences of environmental variables (T + pH + O2 + EC + SPM + NO2 + NO3 + NH4 + POC + DOC + PO4 + Alkalinity), distance and season on the bacterial community variation using variation partitioning analyses [42], using the varpart function in the R package ‘vegan’ package.

## Results and Discussion

### Physico-chemical and Biological Characterization of the Zenne River Samples

**Figure 2:**
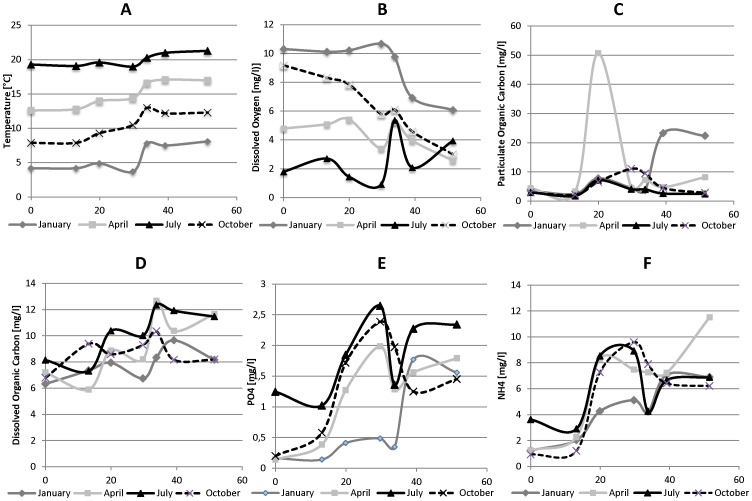
Longitudinal profiles of some physico-chemical parameters. (**A**) Temperature; (**B**) Dissolved Oxygen; (**C**) Particulate organic carbon (POC); (**D**) Dissolved organic carbon (DOC) (**E**) PO_4,_ (**F**) NH_4_.

The four sampling campaigns were characterized by very contrasting temperatures (Fig. 2A), covering a range from 4°C (January) to 22°C (July). An increase of the temperature is systematically observed after the discharge of WWTPs effluents regardless of the season. This effect is particularly marked after Brussels-North WWTP considering its high volumes of effluent discharge into the river.

In fall and winter, the amount of dissolved oxygen (DO, Fig. 2B) in water was quite high at the entrance of the study zone and decreased in the Brussels area. In winter a rapid decrease was observed after the discharge of the Brussels North WWTP, whereas in autumn the decrease was continuous all along the river. In contrast, in summer, DO was already very low at the entrance of the study zone (around 2 mg L^−1^) and dropped to 1 mg L^−1^ in the Brussels area values which are below the lethal concentration for fish [43], [44]. The release of well aerated effluents from Brussels North WWTP obviously increased the oxygen level in the river that decreased rapidly further downstream.

During our sampling campaigns, the particulate organic carbon (POC) content was detected in the range of 2–11 mg/L in all the stations, except three samples: Z5 in April, (51 mg/L, downstream from the Brussels South WWTP discharge) and Z10 and Z12 in January (23 mg/L, downstream from the Brussels North WWTP discharge). These abnormal increases in POC concentrations seem to be associated in April with the discharge of sludge from the activated sludge process at Brussels South WWTP. Such sludge release often occurs at Brussels South WWTP and was confirmed by microscopic observation for the studied April 2010 situation. In addition, in April, the driest studied period, the water mass at Z5 was very heterogeneous (due to the absence of a good mixing between WWTP South effluents and river water) so that particles were overrepresented in this sample. In January, the increase in POC downstream from Brussels area seems to be due to some untreated wastewater released into the river from station Z10. A significant increase in the ratio POC/SPM at Z10 and Z12 stations (25 to 30% C compared to 15% in Z9) was an indication of a high release of untreated sewage (with SPM rich in organic carbon) upstream from station Z10. On the other hand, DOC content was detected in the range of 5–13 mg/L in all the sampling stations. An increase in DOC concentration along the river was observed (Fig. 2D). Furthermore, in all the parameters measured, only NH_4_ and PO_4_ were positively correlated with each other, regardless of sampling season. These two parameters had a peak concentration downstream from the Brussels South WWTP (Z5) during each sampling campaign, indicating a major load of NH_4_ and PO_4_ from this WWTP effluent, due to the lack of tertiary treatment in Brussels South WWTP (Fig. 2E and 2F).

The total number of bacteria enumerated by epifluoresence microscopy ranged from 2.2×10^9^ to 2.3×10^10^ cells/L (data not shown). These bacterial abundances are in the upper range of the values reported in freshwaters but abundances of bacteria higher than 10^10^ cells/L were already reported in rivers receiving high amounts of wastewaters [45]. Although ANOVA analysis revealed no significant difference between seasons, median values in January and April were higher than in July and October.

### Bacterial Community Structure and Composition in the Zenne River and WWTPs Effluents Samples

After denoising and chimera checking, a total of 80109 sequences, with a mean length of 378 bp, were obtained for further analysis from the 28 samples of the Zenne River (all stations and seasons together) and 21114 from the 8 samples of treated wastewaters (4 from Brussels North WWTP and 4 from Brussels South WWTP).

First of all, the data from river samples were compared to those from wastewater effluents, regardless of sampling time and station. Richness was estimated to be 1786 and 4407 OTUs_0.03_ for WWTPs effluents and the river, respectively, while Chao indices were found to be more than 2000 for WWTPs samples, and 4500 for the river samples (Table 1). The rarefaction curves at 0.03 (Fig. S1A) showed that the global diversity of the two series of samples was well represented with the number of sequences analyzed. A recent study done on different treated wastewater effluents reported that species richness might vary between 1500 and 4000 based on the type of the treatment applied [46]. Detection of lower bounds for species richness of WWTPs effluents in comparison to river samples might be due the massive enrichment of some species which are very well adapted to the conditions encountered in some wastewater treatment processes such as activated sludge. When the samples were analyzed individually, richness was estimated between 573 and 1073 in January (Fig. S1B). In April, samples reached to the saturation between 132 and 1271, increase in richness was observed after WWTPs discharges (Fig. S1C). In July, no sample except Z3 has reached to saturation (Fig. S1D). Richness was estimated between 349 and 774 in October (Fig. S1E). The highest richness in WWTPs effluents was detected in Brussels North in January (Fig. S1F).

**Table 1:** Observed bacterial richness and diversity estimates from analyzed Zenne River [28 samples] and WWTPs [8 samples] samples.

Samples	Cutoff	N	Richness	Chao	LCI95	UCI95	H’	varH
WWTPs*	0.03	22126	1816	2113	2051	2192	6.17	0.00012
WWTPs	0.05	22126	1366	1538	1494	1598	5.84	0.00012
Zenne River*	0.03	80188	4408	4551	4521	4590	6.72	0.00004
Zenne River	0.05	80188	3000	3073	3053	3101	6.23	0.00004

N:Number of sequences.

H’: Shannon Index.

varH: Shannon index variance.

LCI:95% lower confidential interval.

UCI:95% upper confidential interval.

Cutoff 0.03– Species level, 0.05– Genus level.

*Only data at species level was further discussed in detail.

**Figure 3:**
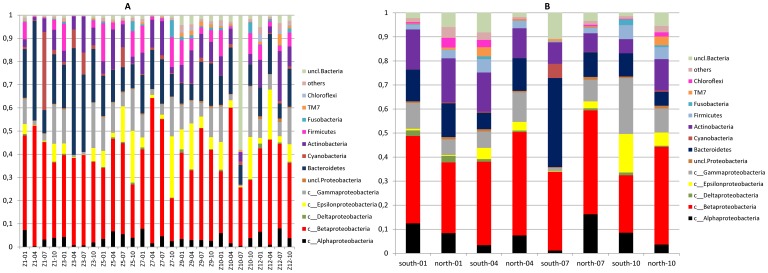
Taxonomic classification of bacterial reads at the phylum level. (**A**) River (28 samples of the Zenne River from all stations and seasons together) and (**B**) wastewater treatment plant effluents.

Figure 3 shows the taxonomic assignations at phylum/class level for all the 28 river samples together (A), and the 8 treated wastewaters samples (B). In the river (Fig. 3A), the dominant phylum was the *Proteobacteria* with up to 73% of the sequences, followed by *Bacteroidetes* up to 46%, Cyanobacteria up to 33%, *Actinobacteria* up to 21% and *Firmicutes* up to16%. Among the *Proteobacteria*, the most abundant class was the *Betaproteobacteria* (up to 63%), followed by the *Epsilonproteobacteria* (up to 26%) G*ammaproteobacteria* (up to 22%), and *Alphaproteobacteria* (up to 8%). Previous studies also reported the dominance of *Proteobacteria* in rivers [7], [28] and the class *Betaproteobacteria* is known to be a typical freshwater clade [47]. At genus level (data not shown), 18% of the sequences remained unclassified. The most abundant genus was: *Flavobacterium* (*Bacteriodetes*) that represented 13% of the sequences, followed by *Arcobacter* and *Malikia* (both 7%, *Epsilon-* and *Betaproteobacteria,* respectively) and *Rhodoferax* (4%, *Betaproteobacteria*). Sixteen percent of the total sequences corresponded to genera that represented less than 1% of the sequences from the river samples.

Concerning the treated wastewater effluents (Fig. 3B), at phylum level the global composition of the bacterial community was very similar to the one observed in the river samples. The dominant phylum was also the *Proteobacteri*a with up to 74% of the sequences, followed by *Bacteroidetes* (up to 37%), *Actinobacteria* (up to 18%). Among the *Proteobacteria*, the most abundant class was the *Betaproteobacteria* (up to 43%), followed by the *Alpha- and Epsilonproteobacteria* (up to 16%), G*ammaproteobacteria* (up to 23%). Although community structures of river and wastewater effluents samples were pretty similar at phylum level, BCC of the treated effluents and the upstream samples were found significantly different at the genus level (R = 0.472, p<0.05). Therefore, after the release of the WWTPs effluent to the river, the BCC of the river might alter significantly. Recently, Drury et al. [48] studied BCC of rivers sediments and reported that WWTP effluents had impact on bacterial community structure and contributed to a biotic homogenization of rivers with different chemical and physical characteristics.

### Multivariate Analysis of the Bacterial Community of All the River Samples

**Figure 4:**
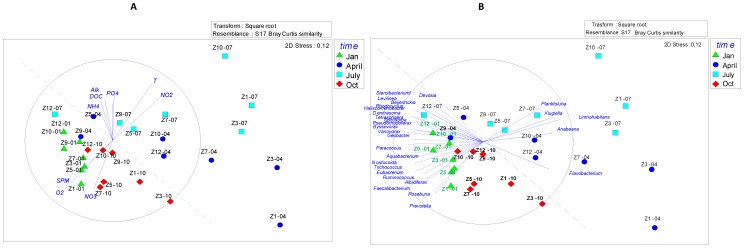
Nonmetric multidimensional scaling (MDS) plot. Environmental variables (**A**) and genera (**B**) are plotted as correlations with the river samples studied. Increasing distance between points equates to decreasing similarity in BCC. MDS plot are based on Bray-Curtis distances generated from square root transformed data. Analysis was conducted using Primer 6. Abbreviations: Jan-January, Oct-October.

MDS analysis at OTU_0.03_ and genus level was performed to see the BCC changes along the river at the different seasons (Fig. 4). Procrustes analyses showed that bacterial diversity patterns were strongly reproducible at both taxonomic levels (correlation coefficient = 0.75, *p* = 0.001) Despite the expected important influence of WWTPs discharge, the two major clusters based on the observed BCC corresponded to, on one hand, the spring and summer samples and, on the other hand, the autumn and winter samples. Previous studies on different river systems also showed large seasonal differences in BCC and bacterial production [49], [50].

In addition, the samples from spring and summer are more scattered than those from autumn and winter suggesting more important variations along the course of the river in the warmer months.

The environmental parameters were shown to be significantly correlated with the samples distribution (Fig. 4A). Temperature was found to be the strongest parameter driving the BCC as already reported in previous studies [50]. Other important parameters were: oxygen concentration, DOC, the SPM content and inorganic nitrogen (NH_4_, NO_2_ and NO_3_) concentrations. These results suggest that the marked seasonality of bacterial community could be associated with *in situ* bacterial activities (DOC and POC biodegradation associated with oxygen consumption and N transformation) which are higher in spring and summer. Indeed, BCC in April and July were positively correlated with PO_4_, NH_4_, total alkalinity and negatively with oxygen_,_ showing active associated respiration processes, whereas opposite interactions were demonstrated in January and October. *In situ* bacterial activities in the river are lower in the colder situations; therefore the input of allochthonous bacteria by WWTPs effluents had a stronger impact on the BCC of the river in fall and winter.

Furthermore, clear association between the phylotypes and the samples were shown (Fig. 4B). An important number of taxa were associated with autumn and winter samples while a lower number were associated with spring and summer samples. Four out of the five genera which were significantly correlated with the spring and summer samples: *Planktoluna, Limnohabitans, Flavobacterium, Anabaena*, have been clearly identified as planktonic bacteria autochthonous from freshwaters [51]. Among the numerous genera present in winter and autumn samples, several belong to groups associated with sludge and wastewaters (*Faecalibacterium, Haliscomenobacter, Beijerinckia, Rhodocyclus, Prevotella, Roseburia, Nostocoida*).

This supports the hypothesis of a higher importance of *in situ* microbial processes in spring and summer, carried by taxa well adapted to the environmental conditions in the river, while allochthonous bacteria associated with wastewaters were found dominant during winter and autumn.

To estimate the relative importance of environmental, seasonal and spatial components in shaping bacterial community structure, we used a variation partitioning analysis. The percentages of the total variation in the bacterial matrix that can be attributed to the different components of variation (environmental, spatial, seasonal and unexplained) were based on the adjusted (unbiased) fractions. When considering the total bacterial diversity, the highest part of the variation (65%) was remained unexplained. The highest part of variation was explained by environmental factors (29%). Despite the important discharges of wastewaters along the river and the significant modification of the river BCC due to the allochthonous bacteria inputs, followed by the strict influence of the sampling location and season (3% each); Besides, correlation between OTUs_0.03_ and environmental parameters were calculated (rho>0.8, p<0.05), and POC, COD and SPM showed significant correlations with some species. For instance, *Acinetobacter, Rhodococcus corprophilus, Thauera* were found to have a linear relationship with POC (Table 2). Such correlations were not previously shown at a lower taxonomic level. However, a significant correlation between *Bacteriodetes* and POC was also previously shown in a study conducted in the English Channel [52].

**Table 2:** Pearson’s correlation coefficients between environmental parameters and OTUs_0.03_.

Environmentalparameter	OTU_0.03_	Assignation	coeffiecient
COD	Otu0032	p_Actinobacteria(100);c_Actinobacteria(100);o_Actinomycetales(100);	0.82
POC	Otu0032	p_Actinobacteria(100);c_Actinobacteria(100);o_Actinomycetales(100);	0.89
POC	Otu0053	p_Actinobacteria(100);c_Actinobacteria(100);o_Actinomycetales(100);f_Nocardiaceae(100);g_Rhodococcus(100);s_Rhodococcus coprophilus(100);	0.85
COD	Otu0119	p_Proteobacteria(100);c_Gammaproteobacteria(100);o_Pseudomonadales(100);f_Moraxellaceae(100);g_Acinetobacter(100);	0.84
POC	Otu0119	p_Proteobacteria(100);c_Gammaproteobacteria(100);o_Pseudomonadales(100);f_Moraxellaceae(100);g_Acinetobacter(100);	0.88
POC	Otu0156	p_Proteobacteria(100);c_Betaproteobacteria(100);o_Rhodocyclales(100);f_Rhodocyclaceae(100);g_Thauera(100);	0.81
SPM	Otu0183	k_Bacteria(100)	0.80
COD	Otu0206	p_Bacteroidetes(100);c_Flavobacteria(100);o_Flavobacteriales(100);f_Flavobacteriaceae(100);g_Flavobacterium(100)	0.83
POC	Otu0206	p_Bacteroidetes(100);c_Flavobacteria(100);o_Flavobacteriales(100);f_Flavobacteriaceae(100);g_Flavobacterium(100)	0.86
SPM	Otu0206	p_Bacteroidetes(100);c_Flavobacteria(100);o_Flavobacteriales(100);f_Flavobacteriaceae(100);g_Flavobacterium(100)	0.81
COD	Otu0303	p_Proteobacteria(100);c_Gammaproteobacteria(100);o_Pseudomonadales(100);f_Moraxellaceae(100);g_Acinetobacter(100)	0.81
POC	Otu0303	p_Proteobacteria(100);c_Gammaproteobacteria(100);o_Pseudomonadales(100);f_Moraxellaceae(100);g_Acinetobacter(100)	0.89
COD	Otu0410	k_Bacteria(100)	0.81
POC	Otu0410	k_Bacteria(100)	0.81
COD	Otu0436	p_Bacteroidetes(100);	0.86
POC	Otu0436	p_Bacteroidetes(100);	0.93
COD	Otu0532	k_Bacteria(100)	0.82
POC	Otu0532	k_Bacteria(100)	0.86
POC	Otu0596	k_Bacteria(100)	0.84
POC	Otu0827	p_Proteobacteria(100);c_Betaproteobacteria(100);	0.80
COD	Otu0926	p_Bacteroidetes(100)	0.83
POC	Otu0926	p_Bacteroidetes(100)	0.83
POC	Otu1079	p_Proteobacteria(100);c_Betaproteobacteria(100);o_Burkholderiales(100);	0.84
POC	Otu1130	p_Actinobacteria(100);c_Actinobacteria(100);o_Actinomycetales(100);	0.84

COD: Chemical Oxygen Demand, POC : Particulate Organic Carbon, SPM:Suspended Particulate Matter.

### Spatio-temporal Variations of Bacterial Community Structure

#### Seasonal fluctuations of bacterial community

In this section, all the samples from each sampling campaign have been grouped and the four groups have been compared in order to evaluate the seasonal impact.

**Figure 5:**
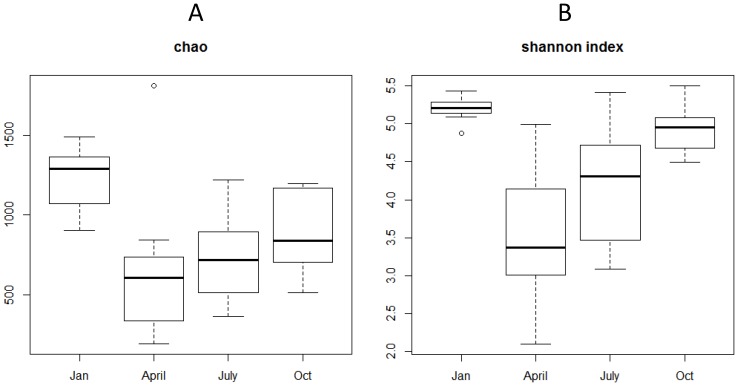
Box plot of richness and diversity estimates per season. (**A**) CHAO and (**B**) Shannon diversity index (All sampling stations of each campaign grouped). Abbreviations: Jan-January, Oct-October.

Figure 5 presents box plots of Chao and Shannon indices for the different seasons (all sampling stations of each campaign grouped). The median of both parameters was the lowest in April; and a gradually increasing trend was observed from April to January (Fig. 5A). The richest and most diverse samples were those from January. Besides, in January the Shannon index had very low standard deviation, while the highest deviation was observed in April (Fig. 5B).

The medians of Chao indices ranged from 600 (April) to 1300 (January), it means that more than twice different species are expected to be found in January with regards to April. The Shannon index also varied a lot ranging from 3.4 (April) to 5.2 (January). There are different seasonal studies done in the aquatic environments showing higher richness estimations in winter [53], [54]. Due to the lower bacterial activity in colder situations, species are more evenly distributed in colder season than in the warmer seasons. However, temperature cannot be the only parameter explaining the difference in diversity indices. Combination of different parameters such as oxygen, organic carbon and/or nitrogen components might have influenced the richness.

Accordingly, effect of seasonal variation was also observed on BCC. ANOSIM analysis (Table 3) revealed that the BCC of the samples from each season were significantly different from each other with the exception of BCCs in April and July which did not differ significantly. Besides, the SIMPER analysis was used to determine the percentage of similarity between the four sampling campaigns in the upstream stations (Z1 and Z3), in order to test if there was any seasonal impact on the BCC of upstream waters before receiving Brussels WWTPs effluents. SIMPER analysis also revealed which individuals contributed most to the dissimilarity between samples (Table S1). The data showed that BCC of the upstream samples in cold seasons (January and October) had the highest similarity with 62%, followed by similarity between April and July with 53%. On the contrary, the highest difference in the BCC was observed between January and April with 63%. Bloom of *Malikia* and *Flavobacterium* in April and relatively high abundant taxa such as *Dechloromonas, Lepthothrix, Pseudomona*s and *Arcobacter* in January explained the difference between the BBC of the two seasons.

**Table 3:** ANOSIM test for differences between time groups**.**

Groups	Global R	p
**January/April**	**0.433**	**0.008**
**January/July**	**0.489**	**0.003**
**January/October**	**0.452**	**0.003**
**April/July**	0.011	0.405
**April/October**	**0.374**	**0.01**
**July./October**	**0.459**	**0.003**

#### Impact of WWTPs effluents on the river BCC

As already mentioned, WWTPs effluents have not the same characteristics in terms of BCC than the receiving water body; therefore, it is important to evaluate the impact of WWTPs effluents on the BCC of the river. First of all, we analyzed if the season has an impact on the BCC of the WWTPs’ effluents. Based on the richness and diversity analysis, Brussels South WWTP had clearly the lowest richness and diversity index in July. Besides, BCC analysis revealed that Brussels South WWTP in July clustered apart from other WWTP effluents (Fig. S2). ANOSIM revealed no further clear separation between the WWTPs effluents based on the season or WWTP (p>0.05).

Figure S3 presents the longitudinal profiles of the Chao and Shannon indexes, each point representing the median of the four sampling seasons. The richness increased after the release of Brussels North WWTP, the standard deviations were large for all the sampling stations, reflecting the important seasonal variations. The diversity (Shannon index) slightly increased after the release of both WWTPs and these stations located just downstream from the WWTPs presented lower standard deviations (indicating lower seasonal variations) than the other sampling stations. To evaluate the impact of WWTPs discharge, three artificial groups of samples were compared: **(1)** the 8 samples of treated wastewaters; **(2)** the 8 river samples collected upstream from Brussels South WWTP discharge (Z1 and Z3 stations from the four sampling campaigns); **(3)** the 20 river samples collected downstream from Brussels South WWTP discharge (5 samples per sampling campaign). First of all, the richness estimation did not change significantly after the discharge, although significant effect of WWTPs discharges on the BCC in the water column was observed. In contrast, a recent study showed that WWTP effluent significantly reduced bacterial diversity and richness in rivers sediments [48], which might indicate a different response of BCC to the WWTP discharges in the water column and the sediments. It has also been previously reported that the community structure of free living bacteria and particle-attached bacteria were different [16]. Due to the settling of the bacteria attached to particulates, the impact of WWTPs effluents on the bacterial richness in the water column and the sediments might be different.

Besides, ANOSIM analysis (Table 4) revealed that upstream samples were significantly different from downstream and WWTP effluents, while no significant differences were found between WWTPs effluents and the downstream samples. SIMPER analysis (Table S2) shows that no single genus can explain more than 5% of the differences. This means that the differences between the upstream and downstream samples are due to a shift after the discharge of very diverse bacterial community from the WWTPs. The impact of WWTPs on the BCC was studied further per season.

**Table 4:** ANOSIM test for differences between location groups.

Groups	Global R	p
**wwtp/upstream**	**0.448**	**0.005**
**wwtp/downstream**	0.119	11.7
**upstream/downstream**	**0.45**	**0.003**

#### Longitudinal profiles of BCC in the river at different seasons

As January and April presented the most contrasting seasonal impact on the bacterial community, they are discussed in more detail below. Longitudinal profiles of BCC in July and October are further discussed in Results S1 and their dendrograms were illustrated in Figure S4.

**Figure 6:**
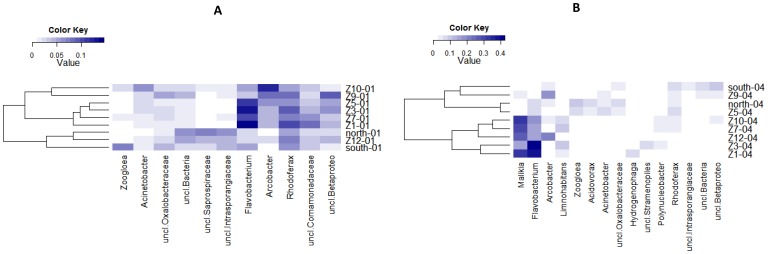
Double hierarchical dendrogram showing the bacterial distribution among the stations. In (**A**) January and (**B**) April. When constructing the heatmaps, differences among sites where characterized using Bray-Curtis distances and differences among taxa were characterized using Euclidean distances. The relative values for bacterial genera are depicted by color intensity.

In January, two major clusters were observed: river samples (Z1 to Z9), on one hand, and wastewater effluents and river sample Z12, in which a release of raw wastewater is suspected, on the other hand (Fig. 6A). River samples had three subclusters: samples collected upstream of the Brussels South WWTP (Z1, Z3); samples collected downstream from the Brussels South WWTP (Z5, Z7) and samples collected downstream from the Brussels North WWTP (Z9, Z10). It is interesting to note that the BCC of the samples taken just downstream from the WWTPs effluents discharge were not dominated immediately by the taxa brought by the effluents. The higher abundance of *Intrasporangiaceae* (closest match to *Tetrasphaera*), *Saprospiraceae* (closest match to *Haliscomenobacter*), and *Rhodoferax* originating from Brussels North WWTPs effluents were only observed at Z12 station. Gram-positive *Tetrasphaera*-related organisms (*Actinobacteria*) are putative polyphosphate-accumulating organisms that are abundant in many full-scale enhanced biological phosphorus removal plants [55] and *Haliscomenobacter* are one of the filamentous bacteria detected in wastewater treatment plants which are responsible for biomass bulking [56], [57]. Their survival in the cold temperature was previously reported [57], [58].

**Figure 7:**
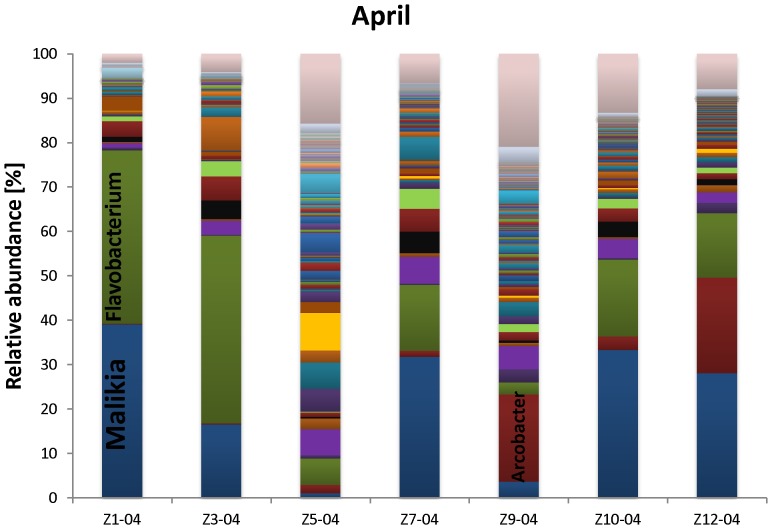
Taxonomic classification of bacterial reads at genus level along the river in April.

In contrast to January, a strong resilience of bacterial communities in the river was observed in April. Bacterial community was strongly modified just downstream from the WWTPs effluents discharges (Fig. 7) and in few kilometers the initial bacterial community composition was recovered and this phenomenon was observed after both WWTPs discharges. This difference between BCC in April and January was also shown by clustering. Two major clusters were observed in April, on one hand, the WWTPs samples and the river samples collected just downstream from the effluents and, on the other hand, the remaining river samples (Fig. 6B). The system seems to be resilient, since the initial situation is recovered already a few kilometers downstream from the discharges of WWTPs (disturbance). SIMPER analysis revealed the dissimilarity between WWTPs and upstream samples 71%. This was due to the bloom of *Malikia* and *Flavobacterium* in the upstream stations and the dominance of *Zooglea, Arcobacter, Acidovorax,* and *Acinetobacter* in the WWTPs’ effluents. *Arcobacter* and *Zooglea* were previously isolated from sewage [59], [60]. *Flavobacteri*a are found in different environment such as soil and freshwater, some of whose species are known to cause disease in freshwater fishes [61] whereas hydrogen-oxidizing and polyhydroxyalkanoate accumulating *Malikia* are restricted to freshwater habitats [62]. We could not find any correlation between *Malikia* bloom and any environmental parameters measured in April. *Malikia* bloom might be due to the low level of river flow at this driest sampling time. Besides, in contrast to January, the impact of WWTPs effluents release has only been observable just downstream from the WWTPs discharges (Z5 and Z9 stations), while the BCC of samples collected further downstream from the WWTPs discharges were similar to the BCC in the upstream part of the river (Fig. 6B). In this case, the composition and structure of bacterial community seems to be dominated by *in situ* processes carried by bacterial groups well adapted to the riverine conditions, with an exception of the sampling points just downstream from the WWTPs discharges.

As it is described above, the impact of WWTPs effluents was season dependent. A recent study showed that sediments of two different rivers with different characteristics in their upstream watershed had almost indistinguishable BCC downstream from the WWTPs discharges, suggesting the potential of WWTPs to reduce the natural variability [48]. Our data suggest that the resilience of the BCC in the water column is season dependent. Studies done by Yannarell et al. [63] and Crump et al. [50] had also contrasting results based on the seasonal impacts on the variability of bacterial communities, which indicates the influence of environmental factors other than temperature like in our case.

## Conclusions

Our data demonstrated that BCC was directly or indirectly influenced by complex seasonal shifts in environmental parameters. Our study showed that disturbance strength has the potential to change the composition of bacterial communities and confirms previous findings that bacterial communities are generally not resistant to disturbances [48], [64], [65]. Indeed, community composition remained altered after the perturbation by WWTPs discharges in three out of the four seasons. However in April, -the driest sampled season -, recovery to pre-disturbance community composition was observed just few kilometers downstream from the WWTPs (pulse disturbance). This is an evidence of the bacterial community resilience in a strongly polluted urban river. Studies dealing with in-situ evidences of freshwater bacterial community stability are scarce and essential for accurate modeling and management of water resources. Environmental and biological factors associated with resilience and resistance must be further studied to assess and prevent the impact of (treated or untreated) sewage pollution on the freshwater ecosystem functioning.

## Supporting Information

Figure S1 Rarefaction analysis for individual and pooled samples of river and wastewater treatment plant at 97% cutoff level.
(PDF)

Figure S2 Longitudinal profiles of CHAO and Shannon index.
(PDF)

Figure S3 The community structure of bacterial genera in wastewater treatment plants. The complete linkage clustering of the samples based on the Bray-Curtis similarity metric demonstrated that, samples were grouped neither based on time nor based on wastewater treatment plant.
(PDF)

Figure S4 Double hierarchical dendrogram showing the bacterial distribution among the stations in [A] July and in [B] October. When constructing the heatmaps, differences among sites where characterized using Bray-Curtis distances and differences among taxa were characterized using Euclidean distances. In addition, data within samples were standardized to facilitate comparisons. The relative values for bacterial genera are depicted by color intensity.
(PDF)

Table S1 Percent of similarity (calculated through SIMPER analysis) between upstream and downstream samples per season.
(XLSX)

Table S2 Results of SIMPER analyses indicating the contribution of specific genus to observed differences in community structure between upstream and downstream samples.
(XLSX)

Results S1 Longitudinal profile of BCC in the river in July and October.
(DOCX)

## References

[1] Abraham WR (2010) Megacities as sources for pathogenic bacteria in rivers and their fate downstream. Inter. J. Microbiol. 2011: 798282. 10.1155/2011/798292 PMC2946570 20885968

[2] United Nations (UN) (2009) Revision of World Urbanization Prospects. www.unpopulation.org.

[3] Kirchman DL (1994) The uptake of inorganic nutrients by heterotrophic bacteria. Microb. Ecol. 28: 255–271. 10.1007/BF00166816 24186453

[4] Kent AD, Yannarell AC, Rusak JA, Triplett EW, McMahon KD (2007) Synchrony in aquatic microbial community dynamics. ISME J 1: 38–47. 18043612 10.1038/ismej.2007.6

[5] Murray AE, Preston CM, Massana R, Taylor LT, Blakis A, et al (1998) Seasonal and spatial variability ofbacterial and archaeal assemblages in the coastal waters near Anvers Island, Antarctica. Appl Environ Microbiol 64: 2585–2595. 9647834 10.1128/aem.64.7.2585-2595.1998 PMC106430

[6] Lindstrom ES, Kamst-Van Agterveld MP, Zwart G (2005) Distribution of typical freshwater bacterial groups is associated with pH, temperature, and lake water retention time. Appl Environ Microbiol 71: 8201–8206. 16332803 10.1128/AEM.71.12.8201-8206.2005 PMC1317352

[7] Ibekwe MA, Leddy MB, Bold RM, Graves AK (2012) Bacterial community composition in low-flowing river water with different sources of pollutants. FEMS Microbiol Ecol 79: 155. 22066546 10.1111/j.1574-6941.2011.01205.x

[8] Araya R, Yamaguchi N, Tani K, Nasu M (2003) Change in the bacterial community of natural river biofilm during biodegradation of aniline-derived compounds determined by denaturing gradient gel electrophoresis Journal of Health Science. 49(5): 379–385.

[9] Echeveste P, Dachs J, Berrojalbiz N, Agusti S (2010) Decrease in the abundance and viability of oceanic phytoplankton due to trace levels of complex mixtures of organic pollutants Chemosphere, 81. (2): 161–168. 10.1016/j.chemosphere.2010.06.072 20673958

[10] Caracciolo BA, Grenni P, Falconi F, Caputo MC, Ancona V, et al (2011) Pharmaceutical waste disposal: assessment of its effects on bacterial communities in soil and groundwater. Chemistry and Ecology 27(1): 43–51.

[11] Huerta B, Marti E, Gros M, López P, Pompêo M, et al.. (2013) Exploring the links between antibiotic occurrence, antibiotic resistance, and bacterial communities in water supply reservoirs. Sci Total Environ. Epub 2013 Apr 13. doi:10.1016/j.scitotenv.2013.03.071. 10.1016/j.scitotenv.2013.03.071 23591067

[12] Shanks OC, Newton RJ, Kelty CA, Huse SM, Sogin ML, et al (2013) Comparison of the microbial community structures of untreated wastewaters from different geographic locales. Appl Environ Microbiol. 79(9): 2906–13. 10.1128/AEM.03448-12 PMC3623150 23435885

[13] Evison LM (1989) Comparative studies on the survival of indicator organisms and pathogens in fresh and seawater. Water Research 20: 309–315.

[14] Garnier J, Billen G, Servais P (1992) Physiological characteristics and ecological rôle of small and large sized bacteria in a polluted river (Seine River, France). Archiv für Hydrobiologie-Ergebnisse Limnologie 37: 83–94.

[15] Okpookwasili GC, Akujobi TC (1996) Bacteriological indicators of tropical water quality. Environmental Toxicology and Water Quality 11: 77–81.

[16] Crump BC, Armbrust EV, Baross JA (1999) Phylogenetic analysis of particle-attached and free-living bacterial communities in the Columbia River, its estuary, and the adjacent coastal ocean. Appl. Environ. Microbiol. 65: 3192–3204. 10.1128/aem.65.7.3192-3204.1999 PMC91474 10388721

[17] Sekiguchi H, Watanabe M, Nakahara T, Xu BH, Uchiyama H (2002) Succession of bacterial community structure along the Changjiang River determined by denaturing gradient gel electrophoresis and clone library analysis. Appl. Environ. Microbiol. 68: 5142–5150. 10.1128/AEM.68.10.5142-5150.2002 PMC126381 12324365

[18] Beier S, Witzel KP, Marxsen J (2008) Bacterial community composition in central European running waters examined by temperature gradient gel electrophoresis and sequence analysis of 16S rRNA genes. Appl. Environ. Microbiol. 74: 188–199. 10.1128/AEM.00327-07 PMC2223207 18024682

[19] Shade A, Chiu CY, McMahon KD (2010) Seasonal and episodic lake mixing stimulate differential planktonic bacterial dynamics. Microb Ecol 59: 546–554. 19760448 10.1007/s00248-009-9589-6

[20] Jaffe R (1991) Fate of hydrophobic organic pollutants in the aquatic environment: a review. Environ Pollut. 69(2–3): 237–57. 10.1016/0269-7491(91)90147-o 15092165

[21] Murray K, Fisher L, Therrien J, George B, Gillespie J (2001) Assessment and use of indicator bacteria to determine sources of pollution to an urban river. J Great Lakes Res 27: 220–229.

[22] Servais P, Garcia-Armisen T, George I, Billen G (2007) Fecal bacteria in the rivers of the Seine drainage network (France): Sources, fate and modelling. Science of the Total Environment 375(1–3): 152–167. 17239424 10.1016/j.scitotenv.2006.12.010

[23] Ouattara NK, Passerat J, Servais P (2011) Faecal contamination of water and sediment in the rivers of the Scheldt drainage network. Environ Monit Assess. 183(1–4): 243–57. 10.1007/s10661-011-1918-9 21336481

[24] Kostanjsek R, Lapanje A, Drobne D, Perović S, Perović A, et al (2005) Bacterial community structure analyses to assess pollution of water and sediments in the Lake Shkodra/Skadar, Balkan Peninsula. Environ Sci Pollut Res Int. 12(6): 361–8. 10.1065/espr2005.07.271 16305142

[25] Li D, Yang M, Li Z, Qi R, He J, et al (2008) Change of bacterial communities in sediments along Songhua River in Northeastern China after a nitrobenzene pollution event. FEMS Microbiol Ecol. 65(3): 494–503. 10.1111/j.1574-6941.2008.00540.x 18616580

[26] Wakelin SA, Colloff MJ, Kookana R (2008) Effect of Wastewater Treatment Plant Effluent on Microbial Function and Community Structure in the Sediment of a Freshwater Stream with Variable Seasonal Flow. Appl Environ Microbiol. 74(9): 2659–2668. 10.1128/AEM.02348-07 PMC2394891 18344343

[27] Zhu J, Zhang J, Li Q, Han T, Xie J, et al.. (2013) Phylogenetic analysis of bacterial community composition in sediment contaminated with multiple heavy metals from the Xiangjiang River in China. Mar Pollut Bull. pii: S0025-326X(13)00079-9. 10.1016/j.marpolbul.2013.02.023 23507235

[28] Kenzaka T, Yamaguchi N, Prapagdee B, Mikami E, Nasu M (2001) Bacterial community composition and activity in urban rivers in Thailand and Malaysia. J. Health Sci. 47: 353–361.

[29] Winter C, Hein T, Kavka G, Mach RL, Farnleitner AH (2007) Longitudinal changes in the bacterial community composition of the Danube River: a whole-river approach. Appl. Environ. Microbiol. 73: 421–431. 10.1128/AEM.01849-06 PMC1796958 17085708

[30] Zhang M, Yu N, Chen L, Jiang C, Tao Y, et al (2012) Structure and seasonal dynamics of bacterial communities in three urban rivers in China. Aquat Sci 74: 113–120.

[31] Brion N, Servais P, Bauwens W, Verbanck M (2012) Past and present chemical and microbiological quality of the Zenne River. In: Wynants and Nuytten (eds) “Bridge over troubled waters”, Crosstalks, VUB press, 241–264.

[32] Porter KG, Feig YS (1980) The use of DAPI for identifying and counting aquatic microflora. Limnol. Oceanog. 25: 943–948.

[33] Llirós M, Casamayor EO, Borrego C (2008) High archaeal richness in the water column of a freshwater sulfurous karstic lake along an interannual study. FEMS Microbiol Ecol. 66: 331–342. 10.1111/j.1574-6941.2008.00583.x 18754782

[34] Callaway TR, Dowd SE, Wolcott RD, Sun Y, Mcreynolds JL, et al (2009) Evaluation of the bacterial diversity in cecal contents of laying hens fed various molting diets by using bacterial tagencoded FLX amplicon pyrosequencing. Poult Sci (88) 298–302. 10.3382/ps.2008-00222 19151343

[35] Smith DM, Snow DE, Rees E, Zischkau AM, Hanson JD, et al (2010) Evaluation of the bacterial diversity of pressure ulcers using bTEFAP pyrosequencing. BMC Med Genomics 3: 41. 20854691 10.1186/1755-8794-3-41 PMC2955665

[36] Edgar RC, Haas BJ, Clemente JC, Quince C, Knight R (2011) UCHIIME improves sensitivity and speed of chimera detection. Oxford Journal of Bioinformatics 27(16): 2194–2200. 10.1093/bioinformatics/btr381 PMC3150044 21700674

[37] Clarke KR, Gorley RN (2006) PRIMER V6: User Manual/Tutorial. Plymouth, UK: PRIMER-E.

[38] Clarke KR, Warwick RM (2001) Change in Marine Communities: An Approach to Statistical Analysis and Interpretation, PRIMERE Ldt, Plymouth, UK.

[39] R Development Core Team (2011) R: A Language and Environment for Statistical Computing. R Foundation for Statistical Computing, Vienna, Austria. ISBN 3-900051-07-0.

[40] Oksanen J, Blanchet FG, Kindt R, Legendre P, O’Hara RB, et al.. (2011) vegan: Community Ecology Package. R package version 1.17–8.

[41] David R (2010) labdsv: Ordination and Multivariate Analysis for Ecology. R package version 1.4–1.

[42] Peres-Neto PR, Legendre P, Dray S, Borcard D (2006) Variation partitioning of species data matrices: estimation and comparison of fractions. Ecology 87: 2614–2625. 17089669 10.1890/0012-9658(2006)87[2614:vposdm]2.0.co;2

[43] Weithman AS, Haas MA (1984) Effects of dissolved-oxygen depletion on the rainbow trout fishery in Lake Taneycomo, Missouri. Trans. Am. Fish. Soc. 113: 109–124.

[44] Ademoroti CMA (1996) Mini water Development in Ibadan. Environmental Chemistry and Toxicology 1st Edition Foludex Press Ibadan, Nigeria. 70–121.

[45] Garnier J, Servais P, Billen G (1992b) Bacterioplankton in the Seine River: impact of the Parisian urban effluents. Can. J. Microbiol. 38: 56–64.

[46] Hu M, Wang X, Wen X, Xia Y (2012) Microbial community structures in different wastewater treatment plants as revealed by 454-pyrosequencing analysis. Bioresource Technology 117: 72–79. 22609716 10.1016/j.biortech.2012.04.061

[47] Methé BA, Hiorns WD, Zehr JP (1998) Contrasts between marine and freshwater bacterial community composition: Analyses of communities in Lake George and six other Adirondack lakes. Limnol Oceanogr 43: 368–374.

[48] Drury B, Rosi-Marshall E, Kelly JJ (2013) Wastewater treatment effluent reduces the abundance and diversity of benthic bacterial communities in urban and suburban rivers. Appl Environ Microbiol. 79(6): 1897–905. 10.1128/AEM.03527-12 PMC3592216 23315724

[49] Van der Gucht K, Sabbe K, De Meester L, Vloemans N, Zwart G, et al (2001) Contrasting bacterioplankton community composition and seasonal dynamics in two neighbouring hypertrophic freshwater lakes. Environ. Microbiol. 3: 680–690. 10.1046/j.1462-2920.2001.00242.x 11846758

[50] Crump BC, Hobbie J (2005) Synchrony and seasonality in bacterioplankton communities of two temperate rivers. Limnol. Oceanogr. 50(6): 1718–1729.

[51] Newton RJ, Jones SE, Eiler A, McMahon KD, Bertilsson S (2011) A guide to the natural history of freshwater lake bacteria. Microbiol Mol Biol Rev 75: 14–49. 21372319 10.1128/MMBR.00028-10 PMC3063352

[52] Lamy D, Obernosterer I, Laghdass M, Artigas LF, Breton E, et al (2009) Temporal changes of major bacterial groups and bacterial heterotrophic activity during a Phaeocystis globosa bloom in the eastern English Channel. Aquat. Microb. Ecol. 58: 95–107.

[53] Gilbert JA, Field D, Swift P, Thomas S, Cummings D, et al (2010) The taxonomic and functional diversity of microbes at a temperate coastal site: a ‘multiomic’ study of seasonal and diel temporal variation. PLoS One 5: e15545. 21124740 10.1371/journal.pone.0015545 PMC2993967

[54] Ghiglione JF, Murray AE (2012) Pronounced summer to winter differences and higher winter time richness in coastal Antarctic marine bacterioplankton. Environmental Microbiology 14(3): 617–629. 22003839 10.1111/j.1462-2920.2011.02601.x

[55] Nielsen PH, Mielczarek AT, Kragelund C, Nielsen JL, Saunders AM, et al (2010) A conceptual ecosystem model of microbial communities in enhanced biological phosphorus removal plants. Water Res 44: 5070–5088. 20723961 10.1016/j.watres.2010.07.036

[56] Eikelboom D (1975) Filementous Organisms observed in activated sludge. Water Research 9: 365–388.

[57] Kotay SM, Datta T, Choi J, Goel R (2011) Biocontrol of biomass bulking caused by Haliscomenobacter hydrossis using a newly isolated lytic bacteriophage. Water Res. 45(2): 694–704. 10.1016/j.watres.2010.08.038 20950835

[58] Finneran KT, Johnsen CV, Lovley DR (2003) *Rhodoferax ferrireducens* sp. nov., a psychrotolerant, facultatively anaerobic bacterium that oxidizes acetate with the reduction of Fe(III). Int. J. Sys. Evol. Microbiol. 53: 669–673. 10.1099/ijs.0.02298-0 12807184

[59] Belanchea L, Valdea JJ, Comas J, Rodab IR, Poch M (2000) Prediction of the bulking phenomenon in wastewater treatment plants. Artificial Intelligence in Engineering 14: 307–317.

[60] Moreno Y, Botella S, Alonso JL, Ferrús MA, Hernández M, et al (2003) Specific Detection of Arcobacter and Campylobacter Strains in Water and Sewage by PCR and Fluorescent *In situ* Hybridization. Appl Environ Microb 69: 1181–1186. 10.1128/AEM.69.2.1181-1186.2003 PMC143587 12571045

[61] Starliper C (2011) Bacterial coldwater diseased fished by *Flavobacterium psychrophilum*. J. Adv. Res. 2(2): 97–108.

[62] Spring S, Wagner M, Schumann P, Kämpfer P (2005) Malikia granosa gen. nov., sp. nov., a novel polyhydroxyalkanoate- and polyphosphate-accumulating bacterium isolated from activated sludge, and reclassification of Pseudomonas spinosa as Malikia spinosa comb. nov. IJSEM 55(2): 621–629. 15774634 10.1099/ijs.0.63356-0

[63] Yannarell AC, Kent AD, Lauster GH, Kratz TK, Triplett EW (2003) Temporal patterns in bacterial communities in three temperate lakes of different trophic status. Microb. Ecol. 46: 391–405. 10.1007/s00248-003-1008-9 12904915

[64] Allison G (2004) The influence of species diversity and tress intensity on community resistance and resilience. Ecological Monographs 74: 117–134 doi:10.1890/02-068

[65] Berga M, Székely A, Langenheder S (2012) Effects of disturbance intensity and frequency on bacterial community composition and function. PLoS ONE 7(5): e36959 doi:10.1371/journal.pone.0036959 22606316 10.1371/journal.pone.0036959 PMC3351442

