# Supplementary material for: Seasonal Variations and Resilience of Bacterial Communities in a Sewage Polluted Urban River
Source: PLoS One. 2014 Mar 25;9(3):e92579. doi: 10.1371/journal.pone.0092579 (PMC3965440; doi:10.1371/journal.pone.0092579)

Figure S1

Rarefaction analysis for pooled and individual samples of river and wastewater treatment plant at 97% cutoff level.

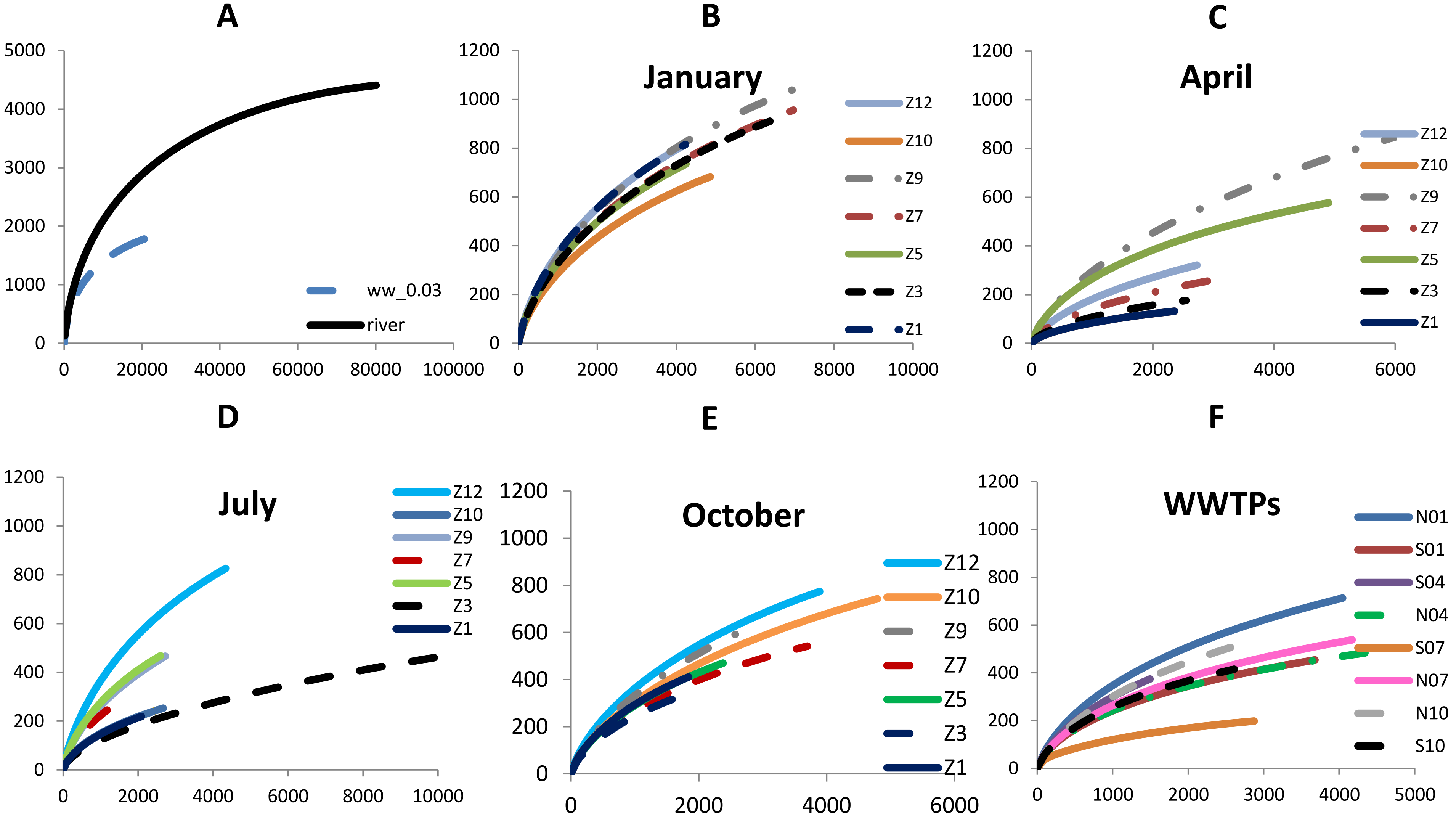

Supplement: Figure S1 — Rarefaction analysis for individual and pooled samples of river and wastewater treatment plant at 97% cutoff level. (PDF) [file pone.0092579.s001.pdf]
