# Supplementary figures and images for: Seasonal Variations and Resilience of Bacterial Communities in a Sewage Polluted Urban River
Source: PLoS One. 2014 Mar 25;9(3):e92579. doi: 10.1371/journal.pone.0092579 (PMC3965440; doi:10.1371/journal.pone.0092579)

**Fig S2** Longitudinal profiles of CHAO and Shannon index

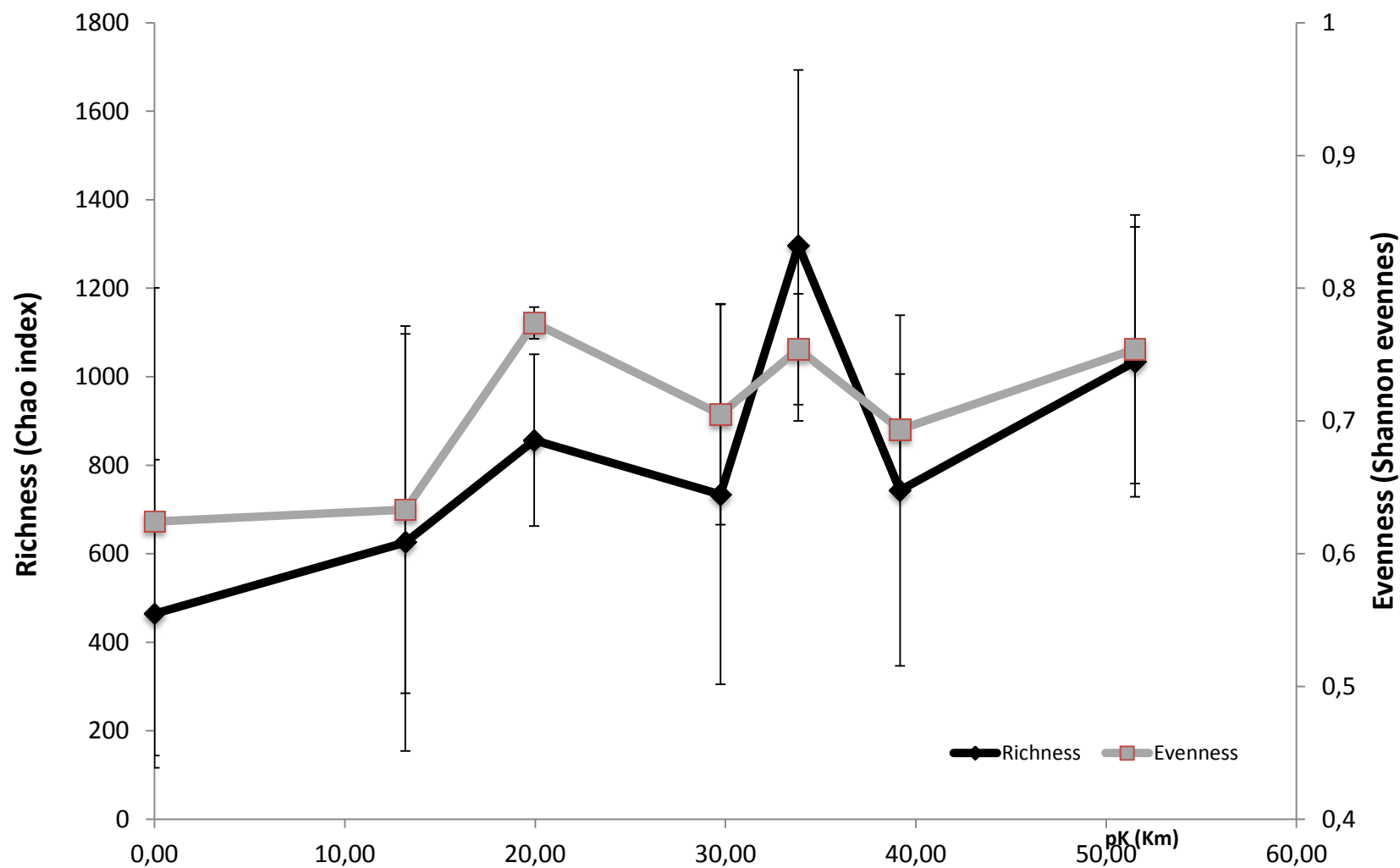

Supplement: Figure S2 — Longitudinal profiles of CHAO and Shannon index. (PDF) [file pone.0092579.s002.pdf]
