# Supplementary material for: Seasonal Variations and Resilience of Bacterial Communities in a Sewage Polluted Urban River
Source: PLoS One. 2014 Mar 25;9(3):e92579. doi: 10.1371/journal.pone.0092579 (PMC3965440; doi:10.1371/journal.pone.0092579)

Figure S3 The community structure of bacterial genera in wastewater treatment plants The complete linkage clustering of the samples based on the Bray-Curtis similarity metric demonstrated that, samples were grouped neither based on time nor based on wastewater treatment plant

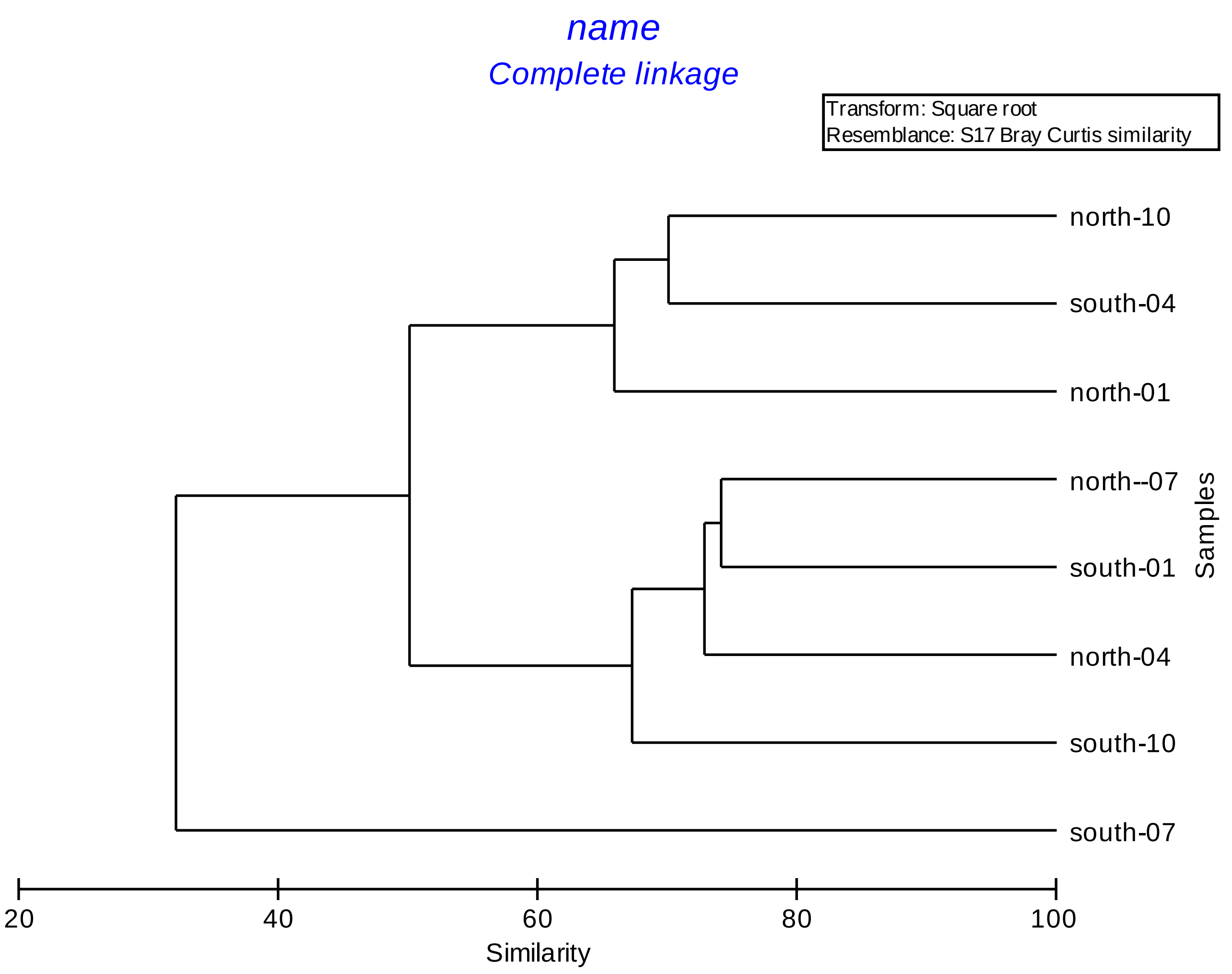

Supplement: Figure S3 — The community structure of bacterial genera in wastewater treatment plants. The complete linkage clustering of the samples based on the Bray-Curtis similarity metric demonstrated that, samples were grouped neither based on time nor based on wastewater treatment plant. (PDF) [file pone.0092579.s003.pdf]
