# Supplementary material for: Seasonal Variations and Resilience of Bacterial Communities in a Sewage Polluted Urban River
Source: PLoS One. 2014 Mar 25;9(3):e92579. doi: 10.1371/journal.pone.0092579 (PMC3965440; doi:10.1371/journal.pone.0092579)

Figure S4 : Double hierarchical dendrogram showing the bacterial distribution among the stations in (A) July and (B) in October. When constructing the heatmaps, differences among sites where characterized using Bray-Curtis distances and differences among taxa were characterized using Euclidean distances. In addition, data within samples were standardized to facilitate comparisons. The relative values for bacterial genera are depicted by color intensity. Genera with a relative abundance higher than 1% are shown.

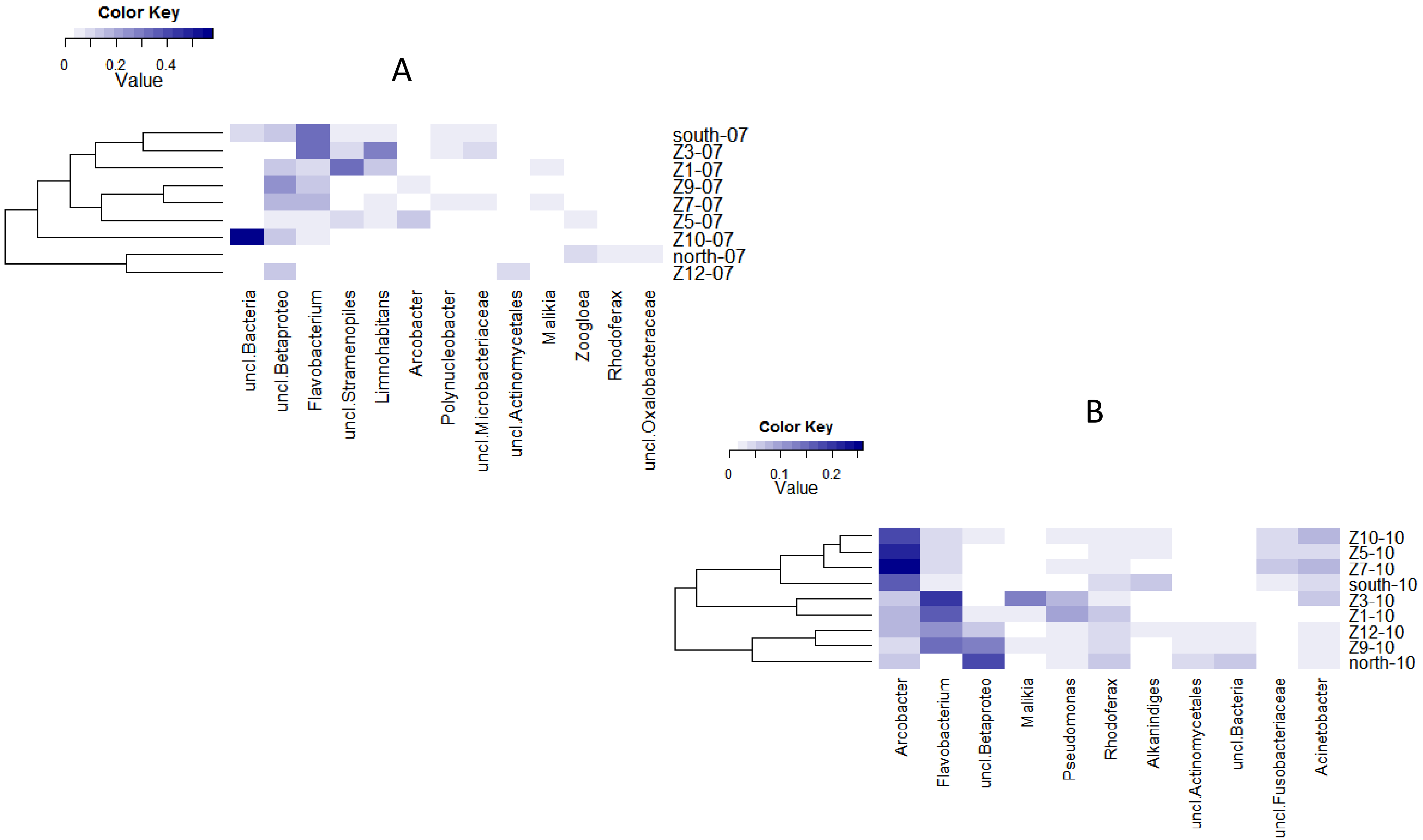

Supplement: Figure S4 — Double hierarchical dendrogram showing the bacterial distribution among the stations in [A] July and in [B] October. When constructing the heatmaps, differences among sites where characterized using Bray-Curtis distances and differences among taxa were characterized using Euclidean distances. In addition, data within samples were standardized to facilitate comparisons. The relative values for bacterial genera are depicted by color intensity. (PDF) [file pone.0092579.s004.pdf]
