# Supplementary material for: Seasonal Variations and Resilience of Bacterial Communities in a Sewage Polluted Urban River
Source: PLoS One. 2014 Mar 25;9(3):e92579. doi: 10.1371/journal.pone.0092579 (PMC3965440; doi:10.1371/journal.pone.0092579)
Supplement: Results S1 — Longitudinal profile of BCC in the river in July and October. (DOCX) [file pone.0092579.s007.docx]

Seasonal variations and resilience of bacterial communities studied by 16S rRNA gene amplicon 454 pyrosequencing in a sewage polluted urban river

Tamara Garcia-Armisen,^1¥^, Özgül İnceoğlu^1¥*^, Nouho Koffi Ouattara^1^, Adriana Anzil^1^, Michel A. Verbanck ^2^, Natacha Brion^3^ and Pierre Servais^1^

***Supplemental Results***

*Longitudinal profile of BCC in the river in July and October*

In July, we have observed three major clustering: (i) upstream and Brussels South WWTP sample, (ii) Z12 and Brussels North WWTP sample and (iii) the other samples (Fig S4A). It was interesting to note that BCC of the Brussels South WWTP sample was highly similar to upstream samples, since *Flavobacterium, Propionivibrio, Limnohabitans* and *Anabaena* were dominating common taxa between the upstream water and the South WWTP. The type strains of all *Limnohabitans* species described so far were all isolated from the water columns of stagnant freshwater systems located in Central Europe (Hahn, 2010). Genus *Anabaena* (cyanobacteria), which synthetizes most of the known toxins, both hepatotoxic (microcystins) and neurotoxic (anatoxin-a(s) and saxytoxins), occurs in freshwater (Carpenter & Carmichael, 1995, Cood et al., 1997) can cause serious problems, possible deoxygenation after important blooms, unpleasant odoriferous substances and the secretion of toxins which present a major health risk (Whitton, 1992). These species lost their dominance downstream from the discharge of Brussels North WWTP effluents, and at Z12; they were mainly replaced by *Dechloromonas, Ornithinicoccus, TM7 and Zooglea.* Some *Dechloromonas* strains degrade oil derivates such as toluene, and might thus contribute to reducing the ecological burden of such aromatic compounds (Chakraborty et al., 2005). Higher abundance of *Dechloromonas* in the Brussels North WWTP may be due to the release of high concentrations of hydrocarbons. *Zooglea* are common soil and water bacteria but they also have the ability to form floc similar to activated sludge under the proper environmental conditions (McKinsey, 2005). *Ornithinicoccus* are known to be soil bacterium (Groth et al., 1999), which theoretically might be introduced to the river from the catchment as dormant cells. TM7 has been detected both in the soil (Borneman et al. 1997) and activated sludge (Bond et al., 1995), however it was not highly abundant in the WWTP sample, which might indicate introduction from the catchment area.

In October, clustering showed the major impact of WWTPs on the BCC (Fig S4B). Three major clusters were observed: (i) upstream samples, (ii) Brussels South WWTP and its downstream samples (Z5, Z7, Z10) and (iii) Brussels North WWTP with the stations located downstream from this WWTP (Z9 and Z12). *Arcobacter, Flavobacterium, Dechloromonas* , which are freshwater and WWTPs related taxa, were the most abundant species in the downstream stations.

**References**

Bond, P. L., P. Hugenholtz, J. Keller, and L. L. Blackall (1995). Bacterial community structures of phosphate-removing and non-phosphate-removing activated sludges from sequencing batch reactors. Appl. Environ. Microbiol.61:1910–1916.

Borneman, J., and E. W. Triplett (1997). Molecular microbial diversity in soils from eastern Amazonia: evidence for unusual microorganisms and microbial population shifts associated with deforestation. Appl. Environ. Microbiol. 63:2647–2653.

Carpenter, E.J. & Carmichael, W.W. (1995). Taxonomy of Cyanobacteria. In: Hallegraeff, G.M.,Anderson, D.M. & Cembella, A.D. (eds.) Manual on harmful marine microalgae. UNESCO,Paris, p.373-380 (IOC manuals and guides, n.33).,

Chakraborty, R.; O’Connor, S. M.; Chan, E.; Coates, J. D. Anaerobic degradation of benzene, toluene, ethylbenzene, and xylene compounds by Dechloromonas strain RCB. Appl. Environ. Microbiol. 2005, 71, 8649–8655.

Cood, G.A., Ward, C.J. & Bell, S.G. (1997). Cyanobacterial toxins: ocurrence, modes of action, health effects and exposure routes. In: Seiler, J.P. & Vilanova, E. (eds.) Archives of Toxicology. Springer-Verlag, Berlin, suppl.19, p.399-410.

Groth I, Schumann P, Martin K, Schuetze B, Augsten K, Kramer I, Stackebrandt E. (1999). Ornithinicoccus hortensis gen. nov., sp. nov., a soil actinomycete which contains L-ornithine. Int J Syst Bacteriol. 4:1717-24.

Hahn, M. W. Kasalický, V., Jezbera, J., Brandt, U., Jezberová, J. & Simek, K. (2010) Limnohabitans curvus gen. nov., sp. nov., a planktonic bacterium isolated from a freshwater lake. International Journal of Systermatic and Evolutionary Microbiology 60: 1358-1365.

McKinsey RE. (2004). Environmental pollution control microbiology. Newyork, USA: Marcel Dekker.

Whitton, B.A. (1992). Diversity, ecology, and taxonomy of the Cyanobacteria. In: Mann, N.H.& Carr, N.G. (eds.) Photosynthetic prokaryotes. Plenum Press, New York, chap.1, p.1-51.
